# Clinical coding of long COVID in English primary care: a federated analysis of 58 million patient records *in situ* using OpenSAFELY

**DOI:** 10.3399/BJGP.2021.0301

**Published:** 2021-08-03

**Authors:** Alex J Walker, Brian MacKenna, Peter Inglesby, Laurie Tomlinson, Christopher T Rentsch, Helen J Curtis, Caroline E Morton, Jessica Morley, Amir Mehrkar, Seb Bacon, George Hickman, Chris Bates, Richard Croker, David Evans, Tom Ward, Jonathan Cockburn, Simon Davy, Krishnan Bhaskaran, Anna Schultze, Elizabeth J Williamson, William J Hulme, Helen I McDonald, Rohini Mathur, Rosalind M Eggo, Kevin Wing, Angel YS Wong, Harriet Forbes, John Tazare, John Parry, Frank Hester, Sam Harper, Shaun O’Hanlon, Alex Eavis, Richard Jarvis, Dima Avramov, Paul Griffiths, Aaron Fowles, Nasreen Parkes, Ian J Douglas, Stephen JW Evans, Liam Smeeth, Ben Goldacre, (The OpenSAFELY Collaborative)

**Affiliations:** The DataLab, Nuffield Department of Primary Care Health Sciences, University of Oxford, Oxford.; The DataLab, Nuffield Department of Primary Care Health Sciences, University of Oxford, Oxford.; The DataLab, Nuffield Department of Primary Care Health Sciences, University of Oxford, Oxford.; Faculty of Epidemiology and Population Health, London School of Hygiene and Tropical Medicine, London.; Faculty of Epidemiology and Population Health, London School of Hygiene and Tropical Medicine, London.; The DataLab, Nuffield Department of Primary Care Health Sciences, University of Oxford, Oxford.; The DataLab, Nuffield Department of Primary Care Health Sciences, University of Oxford, Oxford.; The DataLab, Nuffield Department of Primary Care Health Sciences, University of Oxford, Oxford.; The DataLab, Nuffield Department of Primary Care Health Sciences, University of Oxford, Oxford.; The DataLab, Nuffield Department of Primary Care Health Sciences, University of Oxford, Oxford.; The DataLab, Nuffield Department of Primary Care Health Sciences, University of Oxford, Oxford.; TPP, Leeds.; The DataLab, Nuffield Department of Primary Care Health Sciences, University of Oxford, Oxford.; The DataLab, Nuffield Department of Primary Care Health Sciences, University of Oxford, Oxford.; The DataLab, Nuffield Department of Primary Care Health Sciences, University of Oxford, Oxford.; TPP, Leeds.; The DataLab, Nuffield Department of Primary Care Health Sciences, University of Oxford, Oxford.; Faculty of Epidemiology and Population Health, London School of Hygiene and Tropical Medicine, London.; Faculty of Epidemiology and Population Health, London School of Hygiene and Tropical Medicine, London.; Faculty of Epidemiology and Population Health, London School of Hygiene and Tropical Medicine, London.; The DataLab, Nuffield Department of Primary Care Health Sciences, University of Oxford, Oxford.; Faculty of Epidemiology and Population Health, London School of Hygiene and Tropical Medicine, London.; Faculty of Epidemiology and Population Health, London School of Hygiene and Tropical Medicine, London.; Faculty of Epidemiology and Population Health, London School of Hygiene and Tropical Medicine, London.; Faculty of Epidemiology and Population Health, London School of Hygiene and Tropical Medicine, London.; Faculty of Epidemiology and Population Health, London School of Hygiene and Tropical Medicine, London.; Faculty of Epidemiology and Population Health, London School of Hygiene and Tropical Medicine, London.; Faculty of Epidemiology and Population Health, London School of Hygiene and Tropical Medicine, London.; TPP, Leeds.; TPP, Leeds.; TPP, Leeds.; EMIS Health, Leeds.; EMIS Health, Leeds.; EMIS Health, Leeds.; EMIS Health, Leeds.; EMIS Health, Leeds.; EMIS Health, Leeds.; EMIS Health, Leeds.; Faculty of Epidemiology and Population Health, London School of Hygiene and Tropical Medicine, London.; Faculty of Epidemiology and Population Health, London School of Hygiene and Tropical Medicine, London.; Faculty of Epidemiology and Population Health, London School of Hygiene and Tropical Medicine, London.; The DataLab, Nuffield Department of Primary Care Health Sciences, University of Oxford, Oxford.

**Keywords:** COVID-19, general practice, electronic health records, long COVID, primary health care

## Abstract

**Background:**

Long COVID describes new or persistent symptoms at least 4 weeks after onset of acute COVID-19. Clinical codes to describe this phenomenon were recently created.

**Aim:**

To describe the use of long-COVID codes, and variation of use by general practice, demographic variables, and over time.

**Design and setting:**

Population-based cohort study in English primary care.

**Method:**

Working on behalf of NHS England, OpenSAFELY data were used encompassing 96% of the English population between 1 February 2020 and 25 May 2021. The proportion of people with a recorded code for long COVID was measured overall and by demographic factors, electronic health record software system (EMIS or TPP), and week.

**Results:**

Long COVID was recorded for 23 273 people. Coding was unevenly distributed among practices, with 26.7% of practices having never used the codes. Regional variation ranged between 20.3 per 100 000 people for East of England (95% confidence interval [CI] = 19.3 to 21.4) and 55.6 per 100 000 people in London (95% CI = 54.1 to 57.1). Coding was higher among females (52.1, 95% CI = 51.3 to 52.9) than males (28.1, 95% CI = 27.5 to 28.7), and higher among practices using EMIS (53.7, 95% CI = 52.9 to 54.4) than those using TPP (20.9, 95% CI = 20.3 to 21.4).

**Conclusion:**

Current recording of long COVID in primary care is very low, and variable between practices. This may reflect patients not presenting; clinicians and patients holding different diagnostic thresholds; or challenges with the design and communication of diagnostic codes. Increased awareness of diagnostic codes is recommended to facilitate research and planning of services, and also surveys with qualitative work to better evaluate clinicians’ understanding of the diagnosis.

## INTRODUCTION

Long COVID has been broadly defined as new or persistent symptoms of COVID-19 beyond the acute phase of SARS-CoV-2 infection.^1^ The National Institute for Health and Care Excellence (NICE) have produced guidance on managing the long-term effects of COVID-19 as these symptoms can have a significant effect on a person’s quality of life.^1^ NICE recognise that as long COVID is such a new condition the exact clinical definition and treatments are evolving.

A recent systematic review found a very high prevalence of persisting COVID symptoms after COVID diagnosis.^2^ For symptoms lasting 4–12 weeks 83% of people reported at least one persisting symptom, whereas for symptoms lasting beyond 12 weeks, the proportion was 56%. The reported associated symptoms are numerous, but include fatigue, shortness of breath, cough, smell or taste dysfunction, cognitive impairment, and muscle pain.

NICE developed their definitions and clinical guidelines using a ‘living’ approach based on early data. This means that the guidelines will be continuously reviewed and updated and it is therefore critical to continue studying the long-term effects of COVID-19 as data accrue, and refine the guidelines appropriately. To support this need, long-COVID SNOMED-CT codes (‘diagnostic codes’ listed in Box 1) were developed and released in the UK in November 2020. To support clinical care and implementation of NICE guidance, distinct SNOMED-CT codes were made available by NHS Digital, which distinguish between the length of ongoing symptoms. SNOMED-CT is an international structured clinical coding system for use in electronic health records. Symptoms between 4 and 12 weeks are defined as ‘ongoing symptomatic disease caused by severe acute respiratory syndrome coronavirus 2’, and symptoms continuing beyond 12 weeks as ‘post-COVID-19 syndrome’.^3^ There are also three assessment codes and 10 referral codes relating to long COVID; however, none of these codes explicitly contain the term ‘long COVID’.

**Box 1.** Long-COVID SNOMED-CT codes and terms

**Code type and code**	**Term**
**Diagnostic codes**	
1325161000000102	Post-COVID-19 syndrome
1325181000000106	Ongoing symptomatic disease caused by severe acute respiratory syndrome coronavirus 2

**Referral codes**	
1325021000000106	Signposting to Your COVID Recovery
1325031000000108	Referral to post-COVID assessment clinic
1325041000000104	Referral to Your COVID Recovery rehabilitation platform

**Assessment codes**	
1325051000000101	Newcastle post-COVID syndrome Follow-up Screening Questionnaire
1325061000000103	Assessment using Newcastle post-COVID syndrome Follow-up Screening Questionnaire
1325071000000105	COVID-19 Yorkshire Rehabilitation Screening tool
1325081000000107	Assessment using COVID-19 Yorkshire Rehabilitation Screening tool
1325091000000109	Post-COVID-19 Functional Status Scale patient self-report
1325101000000101	Assessment using Post-COVID-19 Functional Status Scale patient self-report
1325121000000105	Post-COVID-19 Functional Status Scale patient self-report final scale grade
1325131000000107	Post-COVID-19 Functional Status Scale structured interview final scale grade
1325141000000103	Assessment using Post-COVID-19 Functional Status Scale structured interview
1325151000000100	Post-COVID-19 Functional Status Scale structured interview

**Table:** How this fits in

Early case definitions and clinical guidelines have been published to describe long COVID, and clinical codes based on these guidelines were published in late 2020. This study found wide variation in the early use of these codes, by practice, geographic region, and practice electronic health record software. Promotion of the clinical guidance and codes is important for future research and ongoing patient care.

Appropriate coding of long COVID is critical for ongoing patient care, research into the condition, policymaking, and public health resource planning. This study set out to describe the use of long-COVID codes in English primary care since their introduction, in a cohort covering approximately 96% of the English population — those covered by the two largest electronic health record providers, EMIS and TPP (SystmOne). A further aim was to describe the variation of use among general practices, demographic variables, and over time.

## METHOD

### Study design and data sources

A population-based cohort study was conducted that calculated the period prevalence of long COVID recording in electronic health record (EHR) data. Primary care records managed by the GP software providers EMIS and TPP were accessed through OpenSAFELY, an open-source data analytics platform created by the authors on behalf of NHS England to address urgent COVID-19 research questions (https://opensafely.org). OpenSAFELY provides a secure software interface allowing a federated analysis of pseudonymised primary care patient records from England in near real-time within the EMIS and TPP highly secure data environments. Nondisclosive, aggregated results are exported to GitHub (an online code repository) where further data processing and analysis takes place. This avoids the need for large volumes of potentially disclosive pseudonymised patient data to be transferred off-site. This, in addition to other technical and organisational controls, minimises any risk of re-identification.

The dataset available to the platform includes pseudonymised data such as coded diagnoses, medications, and physiological parameters. No free-text data were included. All activity on the platform is publicly logged and all analytic code and supporting clinical coding lists are automatically published. In addition, the framework provides assurance that the analysis is reproducible and reusable. Further details on the information governance and platform can be found in Supplementary Appendix S1.

### Population

All people registered with a general practice on the 1 November 2020 were included.

### Outcome

The outcome was any record of long COVID in the primary care record. This was defined using a list of 15 UK SNOMED-CT codes (Box 1) and categorised as diagnostic (two codes), referral (three codes), and assessment (10 codes).^4^ The outcome was measured between the study start date (1 February 2020) and the end date (25 April 2021). Although the start date is before the codes were created, it is possible for a GP to backdate diagnostic codes in a GP system when they are entered. Timing of outcomes was determined by the first record of a SNOMED-CT code for each person, as determined by the date recorded by the clinician.

### Stratifiers

Demographic variables were extracted including age (in categories), sex, geographic region, Index of Multiple Deprivation (IMD, divided into quintiles), and ethnicity. IMD is a widely used geographical-based measure of relative deprivation based on factors such as income, employment, and education. Counts and rates of recorded events were stratified by each demographic variable. Recording of each SNOMED-CT code was assessed individually, in this case, counting every recorded code including repeated codes, rather than one per patient.

### Statistical methods

Proportions of patients with long-COVID codes over the whole study period per 100 000 patients, 95% confidence intervals (CIs) of those proportions, and the distribution of codes by each stratification variable were calculated. All long-COVID-related codes, as listed in Box 1, were included.

### Software and reproducibility

Data management and analysis was performed using the OpenSAFELY software libraries and Jupyter notebooks, both implemented using Python 3. More details are available in Supplementary Appendix S1. This is an analysis delivered using federated analysis through the OpenSAFELY platform. A federated analysis involves carrying out patient-level analysis in multiple secure datasets, then later combining them: codelists and code for data management and data analysis were specified once using the OpenSAFELY tools; then transmitted securely from the OpenSAFELY jobs server to the OpenSAFELY–TPP platform within TPP’s secure environment, and separately to the OpenSAFELY–EMIS platform within EMIS’s secure environment, where they were each executed separately against local patient data; summary results were then reviewed for disclosiveness, released, and combined for the final outputs. All code for the OpenSAFELY platform for data management, analysis, and secure code execution is shared for review and reuse under open licenses at GitHub. com/OpenSAFELY. All code for data management and analysis for this article is shared for scientific review and reuse under open licenses on GitHub (https://github.com/opensafely/long-covid).

## RESULTS

### Cohort characteristics and overall rate of recording

There were 58.0 million people in the combined cohort in total; 24.0 million in the TPP cohort and 34.0 million in the EMIS cohort. Demographics of the cohort are described in Table 1.

**Table 1.** Characteristics of the cohort

**Characteristic**	**TPP**		**EMIS**		**Combined**	
		
	***n***	**%**	***n***	**%**	***n***	**%**
**Total**	24 011 964	100.0	34 032 530	100	58 044 494	100

**Age group, years**						
0–17	4 821 223	20.1	6 901 845	20.3	11 723 068	20.2
18–24	1 901 509	7.9	2 884 964	8.5	4 786 473	8.2
25–34	3 340 123	13.9	4 962 526	14.6	8 302 649	14.3
35–44	3 220 499	13.4	4 745 812	13.9	7 966 311	13.7
45–54	3 230 861	13.5	4 546 614	13.4	7 777 475	13.4
55–69	4 202 414	17.5	5 697 231	16.7	9 899 645	17.1
70–79	2 080 859	8.7	2 699 998	7.9	4 780 857	8.2
≥80	1 214 476	5.1	1 593 540	4.7	2 808 016	4.8

**Sex**						
Female	12 004 974	50.0	17 014 169	50.0	29 019 143	50.0
Male	12 006 990	50.0	17 018 361	50.0	29 025 351	50.0

**Region**						
East of England	5 638 753	23.5	1 341 520	3.9	6 980 273	12.0
East Midlands	4 191 051	17.5	763 830	2.2	4 954 881	8.5
London	1 702 673	7.1	7 804 070	22.9	9 506 743	16.4
North East	1 100 356	4.6	1 189 619	3.5	2 289 975	3.9
North West	2 067 131	8.6	6 875 180	20.2	8 942 311	15.4
South East	1 582 440	6.6	7 191 261	21.1	8 773 701	15.1
South West	3 304 393	13.8	2 488 558	7.3	5 792 951	10.0
West Midlands	988 286	4.1	5 057 090	14.9	6 045 376	10.4
Yorkshire and The Humber	3 427 713	14.3	1 278 147	3.8	4 705 860	8.1
Missing	9168	0.0	43 255	0.1	52 423	0.1

**IMD quintile**						
1 (most deprived)	4 818 642	20.1	7 015 392	20.6	11 834 034	20.4
2	4 707 307	19.6	7 244 664	21.3	11 951 971	20.6
3	4 941 725	20.6	6 633 133	19.5	11 574 858	19.9
4	4 655 595	19.4	6 401 478	18.8	11 057 073	19.0
5 (least deprived)	4 302 292	17.9	6 635 613	19.5	10 937 905	18.8
Missing	586 403	2.4	102 250	0.3	688 653	1.2

**Ethnicity**						
White	14 573 038	60.7	17 677 690	51.9	32 250 728	55.6
Mixed	319 793	1.3	581 965	1.7	901 758	1.6
South Asian	1 500 012	6.2	2 489 843	7.3	3 989 855	6.9
Black	515 866	2.1	1 173 341	3.4	1 689 207	2.9
Other	476 065	2.0	754 993	2.2	1 231 058	2.1
Missing	6 627 190	27.6	11 354 698	33.4	17 981 888	31.0

*IMD = Index of Multiple Deprivation.*

Up to 25 April 2021, there were 23 273 (0.04%) patients with a recorded code indicative of a long-COVID diagnosis (Table 2). A higher proportion of these recorded diagnoses were in EMIS, with 18 262 (0.05%), compared with 5011 (0.02%) in TPP. Taking into account the larger total number of patients in EMIS practices, the rate over the whole study period was 53.7 per 100 000 people (95% CI = 52.9 to 54.4) in EMIS and 20.9 (95% CI = 20.3 to 21.4) in TPP.

**Table 2.** Counts and rates of long-COVID coding stratified by demographic variable

**Characteristic**	**TPP**			**EMIS**			**Combined**		
		
	**Long COVID, *n***	**Column, %**	**Rate per 100 000**	**Long COVID, *n***	**Column, %**	**Rate per 100 000**	**Long COVID, *n***	**Column, %**	**Rate per 100 000 (95% CI)**
**Total**	5011	100	20.9	18 262	100	53.7	23 273	100	40.1 (39.6 to 40.6)

**Age group, years**									
0–17	94	1.9	1.9	248	1.4	3.6	342	1.5	2.9 (2.6 to 3.2)
18–24	177	3.5	9.3	684	3.7	23.7	861	3.7	18.0 (16.8 to 19.2)
25–34	592	11.8	17.7	2267	12.4	45.7	2859	12.3	34.4 (33.2 to 35.7)
35–44	1033	20.6	32.1	4077	22.3	85.9	5110	22.0	64.1 (62.4 to 65.9)
45–54	1392	27.8	43.1	5183	28.4	114.0	6575	28.3	84.5 (82.5 to 86.6)
55–69	1361	27.2	32.4	4869	26.7	85.5	6230	26.8	62.9 (61.4 to 64.5)
70–79	261	5.2	12.5	693	3.8	25.7	954	4.1	20.0 (18.7 to 21.2)
≥80	101	2.0	8.3	241	1.3	15.1	342	1.5	12.2 (10.9 to 13.5)

**Sex**									
Female	3227	64.4	26.9	11 893	65.1	69.9	15 120	65.0	52.1 (51.3 to 52.9)
Male	1784	35.6	14.9	6369	34.9	37.4	8153	35.0	28.1 (27.5 to 28.7)

**Region^a^**									
East of England	913	18.2	16.2	505	2.8	37.6	1418	6.1	20.3 (19.3 to 21.4)
East Midlands	775	15.5	18.5	314	1.7	41.1	1089	4.7	22.0 (20.7 to 23.3)
London	265	5.3	15.6	5021	27.5	64.3	5286	22.7	55.6 (54.1 to 57.1)
North East	328	6.5	29.8	628	3.4	52.8	956	4.1	41.7 (39.1 to 44.4)
North West	395	7.9	19.1	4185	22.9	60.9	4580	19.7	51.2 (49.7 to 52.7)
South East	593	11.8	37.5	3463	19.0	48.2	4056	17.4	46.2 (44.8 to 47.7)
South West	797	15.9	24.1	1004	5.5	40.3	1801	7.7	31.1 (29.7 to 32.5)
West Midlands	288	5.7	29.1	2598	14.2	51.4	2886	12.4	47.7 (46.0 to 49.5)
Yorkshire and The Humber	655	13.1	19.1	528	2.9	41.3	1183	5.1	25.1 (23.7 to 26.6)

**IMD quintile**									
1 (most deprived)	912	18.2	18.9	4031	22.1	57.5	4943	21.2	41.8 (40.6 to 42.9)
2	970	19.4	20.6	4383	24.0	60.5	5353	23.0	44.8 (43.6 to 46.0)
3	1049	20.9	21.2	3486	19.1	52.6	4535	19.5	39.2 (38.0 to 40.3)
4	1013	20.2	21.8	3287	18.0	51.3	4300	18.5	38.9 (37.7 to 40.10)
5 (least deprived)	949	18.9	22.1	3034	16.6	45.7	3983	17.1	36.4 (35.3 to 37.5)
Missing	118	2.4	20.1	41	0.2	40.1	159	0.7	23.1 (19.5 to 26.7)

**Ethnicity**									
White	3393	84.8	23.3	7350	74.4	41.6	10 743	46.2	33.3 (32.7 to 33.9)
Mixed	63	1.6	19.7	223	2.3	38.3	286	1.2	31.7 (28.0 to 35.4)
South Asian	392	9.8	26.1	1549	15.7	62.2	1941	8.3	48.6 (46.5 to 50.8)
Black	91	2.3	17.6	560	5.7	47.7	651	2.8	38.5 (35.6 to 41.5)
Other	63	1.6	13.2	193	2.0	25.6	256	1.1	20.8 (18.2 to 23.3)
Missing	1009	20.1	15.2	8387	45.9	73.9	9396	40.4	52.3 (51.2 to 53.3)

a *Missing data redacted due to small numbers in at least one cell (*n *= ≥5). IMD = Index of Multiple Deprivation.*

### Rate of coding stratified by demographics

Counts and rates of long-COVID coding stratified by demographic factors are presented in Table 2. For age, the incidence of long-COVID recording rose to a peak in the 45–54 years age group, before declining again in older age groups. Females had a higher rate of recording than males (52.1 [95% CI = 51.3 to 52.9] versus 28.1 [95% CI = 27.5 to 28.7] per 100 000 people). Counts of long-COVID recording by IMD and ethnicity are reported in Table 2. Also reported in Table 2 are counts broken down by EHR software provider. Here some similarities and differences in the rates were observed; the proportions of events for age and sex are fairly comparable whereas region, IMD, and ethnicity show some differences.

### Geographic and practice distribution of coding

The rate of coding varied substantially between regions (Table 2), from a minimum proportion of 20.3 per 100 000 people in the East of England (95% CI = 19.3 to 21.4) to 55.6 in London (95% CI = 54.1 to 57.1). Given that EMIS practices overall had higher rates of recording than TPP, some of this geographic variation may be related to the EHR software provider. For example, EMIS covers a high proportion of the London population, whereas TPP covers a high proportion of the East of England (Table 1).

**Figure 1.**
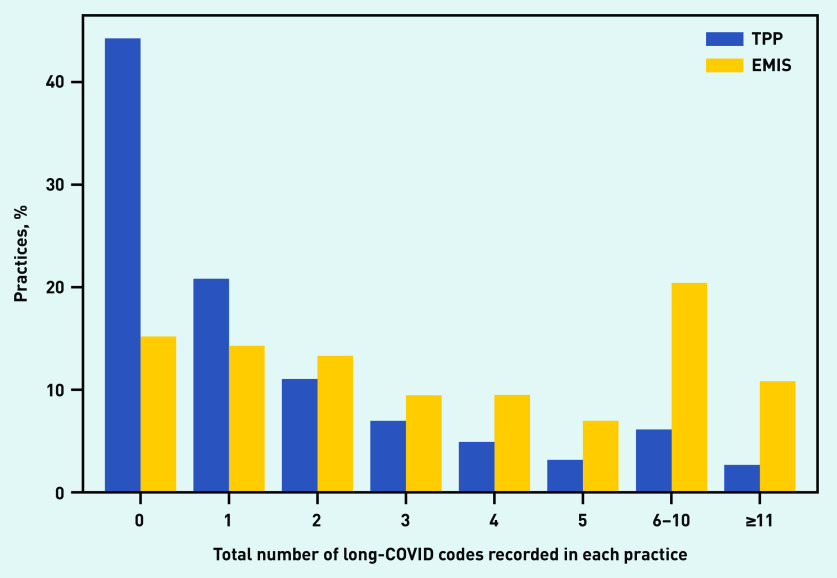
*Volume of code use in individual practices, stratified by the electronic health record provider of the practice (TPP/SystmOne or EMIS).*

Over one-quarter (26.7%) of practices have not used the codes at all (data not shown). This proportion is much higher in practices using TPP (44.2%) than those using EMIS (15.1%) (Figure 1). The distribution is described more fully in Figure 1. The highest number of codes in a single practice was 150 (data not shown).

### Rate of coding over time

**Figure 2.**
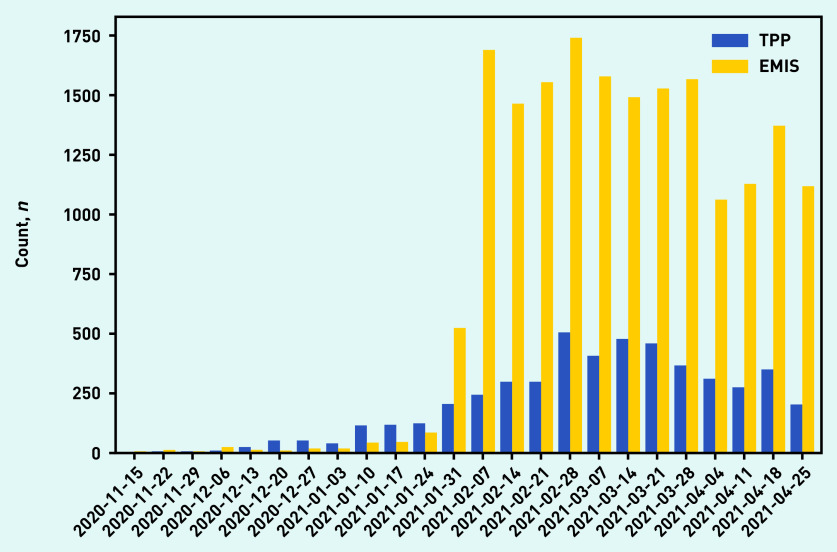
*Use of long-COVID codes over time, stratified by the electronic health record provider of the practice (TPP/SystmOne or EMIS). Reporting lag may affect recent dates.*

The number of recorded events was relatively low until the end of January 2021, after which there was an increase in coding (Figure 2). This increase was more marked in EMIS practices, which before that time had recorded fewer long-COVID codes overall than TPP practices. It was very infrequent to find records that had been backdated to before November 2020 when the codes were created, with <0.1% of codes coded as occurring before November 2020 (data not shown).

### Coding of individual SNOMED-CT codes

The diagnostic codes were the most commonly used codes, particularly the ‘Post-COVID-19 syndrome’ code, which accounted for 64.3% of all recorded codes (Table 3). There were differences in the distribution of codes, however, between TPP and EMIS practices. Codes relating to assessment of long COVID accounted for just 2.4% of long-COVID codes used to date.

**Table 3.** Total use of each individual long-COVID-related code^a^

**Code type ad code**	**Term**	**Count in TPP/SystmOne practices, *n***	**Count in EMIS practices, *n***	**Total count, *n***	**Percentage of total code use**
**Total**		6516	29 991	36 507	100

**Diagnostic codes**					
1325161000000102	Post-COVID-19 syndrome	1187	22 281	23 468	64.3
1325181000000106	Ongoing symptomatic disease caused by severe acute respiratory syndrome coronavirus 2	1895	1094	2989	8.2

**Referral codes**					
1325021000000106	Signposting to Your COVID Recovery	680	368	1048	2.9
1325031000000108	Referral to post-COVID assessment clinic	1128	5204	6332	17.3
1325041000000104	Referral to Your COVID Recovery rehabilitation platform	1398	408	1806	4.9

**Assessment codes**					
1325051000000101	Newcastle post-COVID syndrome Follow-up Screening Questionnaire	6	300	306	0.8
1325061000000103	Assessment using Newcastle post-COVID syndrome Follow-up Screening Questionnaire	8	90	98	0.3
1325071000000105	COVID-19 Yorkshire Rehabilitation Screening tool	56	93	149	0.4
1325081000000107	Assessment using COVID-19 Yorkshire Rehabilitation Screening tool	129	57	186	0.5
1325091000000109	Post-COVID-19 Functional Status Scale patient self-report	≤5	25	25	0.1
1325101000000101	Assessment using Post-COVID-19 Functional Status Scale patient self-report	≤5	25	25	0.1
1325121000000105	Post-COVID-19 Functional Status Scale patient self-report final scale grade	≤5	13	13	0.0
1325131000000107	Post-COVID-19 Functional Status Scale structured interview final scale grade	0	≤5	0	0.0
1325141000000103	Assessment using Post-COVID-19 Functional Status Scale structured interview	29	22	51	0.1
1325151000000100	Post-COVID-19 Functional Status Scale structured interview	≤5	11	11	0.0

a *This is distinct from Table 2 in that it counts all coded events, including where patients have been coded more than once.*

## DISCUSSION

### Summary

As of late April 2021, 23 273 people had a record of at least one long-COVID code in their primary care record. Use between different general practices varied greatly, and a large proportion (26.7%) have never used any long-COVID codes. Substantially higher recording in practices that use EMIS compared with those that use TPP was found. Among those people who did have a recorded long-COVID code, rates were highest in the working-age population and were more common in females.

### Strengths and limitations

The key strength of this study is its unprecedented scale; >58 million people were included, 96% of the population in England. In contrast with many studies that use EHR data, in this study it was possible to compare long-COVID diagnostic codes between practices that use different software systems. A striking disparity was found: this has important implications for understanding whether clinicians are using the codes appropriately. A key weakness of this data for estimating true prevalence of long COVID in primary care, and factors associated with the condition, is that it relies on clinicians formally entering a diagnostic or referral code into the patient’s record: this is a limitation of all EHR research for all clinical conditions and activity, however, the emergence of a new diagnosis and the recent launch of a new set of diagnostic codes may present challenges in this regard. As a result of these current limitations, this study did not aim to estimate the prevalence of long COVID, or aim to make causal inferences about the observed variation.

### Comparison with existing literature

To the authors’ knowledge, there are no other studies on prevalence of long COVID using clinicians’ diagnoses or EHRs data. There are numerous studies using self-reported data from patients on the prevalence of continued symptoms following COVID-19, with estimates varying between 4.5% and 89%, largely because of highly variable case definitions;^5^ individual symptoms characterising long COVID have been reported as fatigue, headache, dyspnoea, and anosmia.^6^ The Office for National Statistics COVID Infection Survey estimates prevalence of self-diagnosed long COVID at 13.7%.^7^ Separately, numerous cohort studies have reported an increased risk of serious cardiovascular and metabolic outcomes following hospital admission with COVID-19,^8^^,^^9^ and there are various prospective studies such as the Post-hospitalisation COVID-19 study following-up patients for the year following their hospital admission.^10^ Other studies have examined variation in clinical coding, with some finding that ‘poor’ coding can lead to altered incidence estimates,^11^ whereas others implicate the design of clinical software systems in influencing variation.^12^^–^14

### Implications for research and practice

The prevalence of long-COVID codes in primary care that are reported in this study is extremely low when compared with current survey data on long-COVID prevalence.^15^^,^^16^ This conflict may be attributable to a range of different possible causes related to information bias including: patients not yet presenting to primary care with long COVID; different clinicians and patients holding different diagnostic thresholds or criteria for long COVID; and issues around coding activity including clinicians not yet knowing about the long-COVID diagnostic codes, the design and text of the long-COVID diagnostic codes, and the design of EHR systems in which the codes can be selected for entry onto a patient record.

The large variation in the apparent rate of long COVID between different geographic regions, practices, and EHR systems strongly suggests that clinicians’ coding practice is inconsistent at present. This suggests variation in awareness of the new diagnostic codes that were only launched in November 2020, and only available in EMIS at the end of January 2021. In addition, the codes for long COVID and associated synonyms do not currently contain the term ‘long COVID’: this was an active choice by NHS Digital who manage SNOMEDCT UK codes.^1^ The October 2020 NICE consultation on management of the long-term effects of COVID-19 does mention the term ‘long COVID’, although the term was not incorporated into the clinical definitions that were translated into diagnostic codes by NHS Digital.^1^ These decisions were carefully thought through at the time they were made; however, as a result of broader contextual shifts in language over time there is now a clear mismatch between formal clinical terminology and popular parlance among clinicians and patients. The view of the authors of this study is that those managing SNOMED-CT terminology for England should either update the long-COVID codes to include the phrase ‘long COVID’, ideally in advance of the upcoming new SNOMED-CT international release; or energetically disseminate their preferred new phrasing to all frontline clinicians, to ensure more appropriate use of these codes. Similarly NICE and other authoritative bodies giving guidance on long COVID should energetically communicate to clinicians the importance of correctly coding long COVID in patient records. It is a high national priority to estimate the prevalence of long COVID, identify its causes and consequences, and plan services appropriately.

The variation in the rate of diagnostic code usage between users of different EHR software is also striking. This difference could plausibly be responsible for some of the other variation described. For example, as noted in the results, some regions have a high percentage of coverage from one software provider. After speaking with clinicians and both software vendors, the reasons for the difference remain unclear, but are likely attributable to differences in user interface, which has previously been shown to influence clinicians’ treatment choices.^13^^,^^14^ This should be addressed by interviewing GPs about their experiences with diagnosing and treating people with long COVID in each system.

Despite these issues around correct *recording* of clinicians’ diagnoses, there also remains a strong possibility that clinicians are not currently *diagnosing* their patients as having long COVID. This may be because patients are not presenting with long COVID to services, for a range of reasons during a pandemic; or their clinicians are not diagnosing them with long COVID when they are seen. The view of the authors is that this can only be resolved by conducting prospective surveys with clinicians themselves, evaluating how many patients they have seen with a condition they would understand to be diagnosable as long COVID, alongside qualitative research on the topic.

The issues with recording of long COVID described here also have implications for future research. It is likely that recording will improve over time, as disease definitions are improved, guidelines are iterated on, and clinicians become more aware of the condition. It is likely also worth considering additional approaches to identifying long COVID in routine medical data. This might include identifying and measuring broad groups of symptoms that are associated with long COVID.^17^

If it is accepted that the different rates of long COVID usage in each subgroup reflects the true comparative risk for each demographic then there are two key findings. First, the lower rate in older patients, despite their higher prevalence of severe acute COVID-19 outcomes,^18^ which may be affected by the competing risk of death in patients with COVID-19. Second, the higher rate of long COVID in females, despite the higher prevalence of severe acute COVID outcomes in males,^18^ which may be explained in part by differences in routine consultation rates between males and females.^19^

In conclusion, current recording of long COVID in primary care is very low, and variable between practices. This may reflect patients not presenting; clinicians and patients holding different diagnostic thresholds; or challenges with the design and communication of diagnostic codes. This analysis will be updated regularly with extended follow-up time.

## References

[1] National Institute for Health and Care Excellence (2020). COVID-19 rapid guideline: managing the long-term effects of COVID-19 NG188.

[2] Domingo FR, Waddell LA, Cheung AM (2021). Prevalence of long-term effects in individuals diagnosed with COVID-19: a living systematic review. medRxiv.

[3] National Institute for Health and Care Excellence (2020). COVID-19 rapid guideline: managing the long-term effects of COVID-19 1 Identifying people with ongoing symptomatic COVID-19 or post-COVID-19 syndrome NG188.

[4] NHS Digital (2020). COVID-19 information standards: COVID-19 SNOMED CT codes by groups 20201221v1.0. https://hscic.kahootz.com/connect.ti/COVID19_info_sharing/view?objectId=67227941.

[5] National Institute for Health Research (2021). Living with Covid19 — Second review.

[6] Sudre CH, Murray B, Varsavsky T (2021). Attributes and predictors of long COVID. Nat Med.

[7] Office for National Statistics (2021). Prevalence of ongoing symptoms following coronavirus (COVID-19) infection in the UK: 1 April 2021.

[8] Ayoubkhani D, Khunti K, Nafilyan V (2021). Post-covid syndrome in individuals admitted to hospital with covid-19: retrospective cohort study. BMJ.

[9] Tazare J, Walker AJ, The OpenSAFELY Collaborative (2021). Rates of serious clinical outcomes in survivors of hospitalisation with COVID-19: a descriptive cohort study within the OpenSAFELY platform. medRxiv.

[10] Evans RA, McAuley H, PHOSP-COVID Collaborative Group (2021). Physical, cognitive and mental health impacts of COVID-19 following hospitalisation — a multi-centre prospective cohort study. bioRxiv.

[11] Tate AR, Dungey S, Glew S (2017). Quality of recording of diabetes in the UK: how does the GP’s method of coding clinical data affect incidence estimates? Cross-sectional study using the CPRD database. BMJ Open.

[12] Tai TW, Anandarajah S, Dhoul N (2007). Variation in clinical coding lists in UK general practice: a barrier to consistent data entry?. Inform Prim Care.

[13] MacKenna B, Bacon S, Walker AJ (2020). Impact of electronic health record interface design on unsafe prescribing of ciclosporin, tacrolimus, and diltiazem: cohort study in English national health service primary care. J Med Internet Res.

[14] MacKenna B, Curtis HJ, Walker AJ (2020). Suboptimal prescribing behaviour associated with clinical software design features: a retrospective cohort study in English NHS primary care. Br J Gen Pract.

[15] Sudre CH, Murray B, Varsavsky T (2020). Attributes and predictors of long-COVID: analysis of COVID cases and their symptoms collected by the Covid Symptoms Study App. medRxiv.

[16] Thompson EJ, Williams DM, Walker AJ (2021). Risk factors for long COVID: analyses of 10 longitudinal studies and electronic health records in the UK. medRxiv.

[17] Al-Aly Z, Xie Y, Bowe B (2021). High-dimensional characterization of post-acute sequelae of COVID-19. Nature.

[18] Williamson EJ, Walker AJ, Bhaskaran K (2020). OpenSAFELY: factors associated with COVID-19 death in 17 million patients. Nature.

[19] Wang Y, Hunt K, Nazareth I (2013). Do men consult less than women? An analysis of routinely collected UK general practice data. BMJ Open.

